# Research Progress of GP4 Protein of Porcine Reproductive and Respiratory Syndrome Virus

**DOI:** 10.3390/vetsci13070718

**Published:** 2026-07-21

**Authors:** Qipeng Zhang, Fang Liang, Jiaman Li, Chen Lv, Huawei Li, Ruining Wang, Mengmeng Zhao, Keshan Zhang

**Affiliations:** 1 School of Animal Science and Technology, Foshan University, Foshan 528225, China; zhangqp1236@163.com (Q.Z.); 15726370117@163.com (F.L.); 13523782338@163.com (J.L.); lc0214fosu@163.com (C.L.); 2 College of Veterinary Medicine, Henan University of Animal Husbandry and Economy, Zhengzhou 450046, China; centrosome@126.com (H.L.); 80882@hnuahe.edu.cn (R.W.)

**Keywords:** porcine reproductive and respiratory syndrome virus, GP4 protein, structural function, genetic evolution

## Abstract

Porcine reproductive and respiratory syndrome, a highly contagious disease, poses a severe threat to the global swine industry. Its causative agent, PRRSV, induces reproductive disorders (abortion, stillbirth) in pregnant sows and respiratory disease in piglets. The host immune system is disrupted by PRRSV, and no specific antiviral drugs are currently available. Thus, vaccination is regarded as the primary strategy for PRRS prevention and control. GP4, a key minor structural protein of PRRSV with an estimated molecular weight of 32 kDa, contains four conserved N-glycosylation sites at residues 37, 84, 120 and 130. Critical neutralizing epitopes are harbored in its extracellular domain (amino acids 40–79). PRRSV binding to the host CD163 receptor is mediated by GP4, which also regulates viral assembly and release, and induces protective immune responses. Hence, GP4 is identified as a central target for PRRS vaccine development. In this review, GP4’s structural characteristics, genetic evolution and interaction mechanisms are summarized. Its roles in viral life cycle, virulence, immune evasion and potential applications are discussed, providing a theoretical reference for PRRSV control.

## Simple Summary

Porcine reproductive and respiratory syndrome (PRRS) is a disease that seriously affects pigs. It causes major losses to the global swine industry. There are no specific antiviral drugs available. This study looks at the roles of the PRRS virus’s GP4 protein to help prevent the disease. GP4 helps the virus infect host cells and triggers immune responses in pigs, and it also affects viral virulence. GP4 can induce protective immune reactions in pigs and could be used for vaccine development. Subunit and vector vaccines may target GP4. Antibodies against GP4 can stop viral infection. GP4 is a key target for controlling PRRS. This research supports the development of safer vaccines and therapeutic antibodies. These will help reduce economic losses and ensure a stable pork supply. This will benefit farmers and the public.

## 1. Introduction

Porcine reproductive and respiratory syndrome (PRRS) was first reported in the United States in the late 1980s, subsequently spreading to continental Europe within approximately three years and further expanding to major swine-producing regions in Asia within five years of its initial emergence [1,2]. Economic impact assessments indicate that this epidemic has resulted in cumulative losses of approximately USD 4 billion to the global swine industry [3]. The primary clinical manifestations of the disease include reproductive disorders in pregnant sows. Pigs of various ages, particularly piglets, exhibit respiratory ailments, often accompanied by viremia, followed by organ failure including cardiac and renal dysfunction [2,4,5].

**Figure 1:**
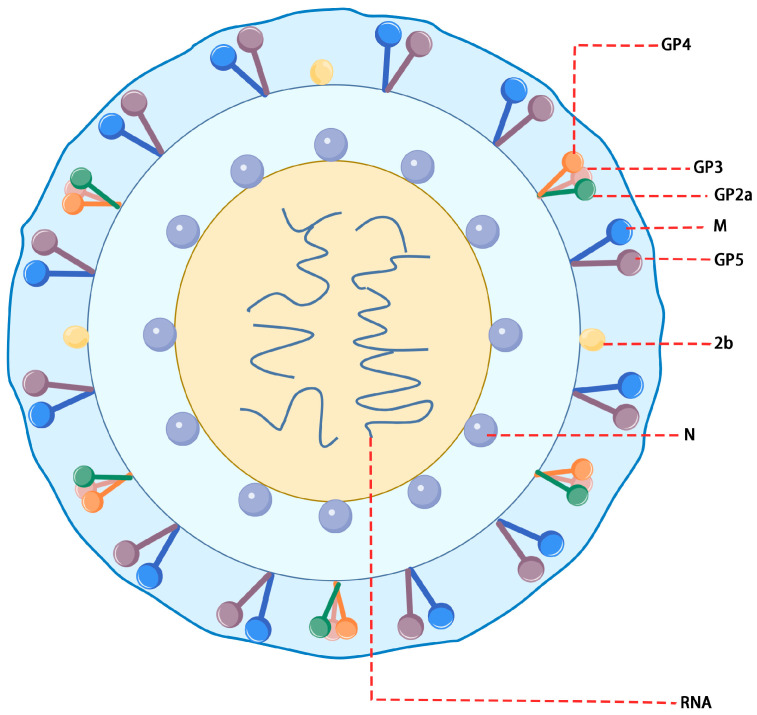
Schematic representation of PRRSV particle structure. The pattern was created with BioGDP [19].

PPRS virus (PRRSV) is a single-stranded, positive-sense RNA virus belonging to the order *Nidovirales*, family *Arteriviridae*, and genus *Arterivirus* [6,7,8]. The virus particles were observed by electron microscopy to be spherical and enveloped, measuring approximately 55–60 nm in diameter, as illustrated in Figure 1. The nucleocapsid size is between 25 and 35 nm [9]. The PRRSV genome is approximately 15 kb in length and contains a capped leader sequence at the 5′ terminus and a poly(A) tail at the 3′ terminus. Variations in the poly(A) tail have been reported to indirectly affect viral virulence [10,11]. The PRRSV genome contains 10 major open reading frames (ORFs) arranged from the 5′ to 3′ terminus, with ORF1a and ORF1b overlapping via ribosomal frameshifting to encode non-structural proteins, and ORF2a, ORF2b, ORF3, ORF4 (encoding GP4), ORF5, ORF5a, ORF6, and ORF7 encoding structural proteins [12,13]. Based on genetic and antigenic differences, PRRSV can be classified into PRRSV-1 and PRRSV-2 [14]. The polyprotein encoded by the ORF1 of PRRSV can be hydrolyzed into 13 nonstructural proteins, specifically Nsp1α, Nsp1β, and Nsp2 through Nsp12 [15,16].

PRRSV major structural proteins have been extensively studied, whereas research on minor structural proteins, including GP4, remains limited [17]. Current investigations of the PRRSV GP4 protein have primarily focused on its structure, functions, and roles in the viral life cycle [18].

This review aims to systematically summarize the structural characteristics, genetic evolution, interaction mechanisms with host and viral proteins, and immunological properties of the GP4 protein, as well as its potential applications in vaccine development, diagnostic reagent production, and therapeutic antibody design, thereby providing a theoretical reference for PRRSV control strategies.

## 2. Molecular Characteristics of GP4 Protein

GP4, an essential structural protein of PRRSV, has been reported to possess a molecular weight of approximately 32 kDa and undergoes extensive glycosylation [20]. GP4 has been shown to contain four glycosylation sites, specifically located at the N-terminal residues 37, 84, 120, and 130. These conserved glycosylation sites are considered essential for its structure and function [21].

In the spatial structure of the GP4 protein, hydrophobic regions are observed at the N-terminus (aa 1–22) and C-terminus (aa 166–183) [22]. The intervening region (aa 23–165) forms the main antigenic domain, with aa 40–79 containing neutralizing epitopes [23]. Specifically, amino acids 40–79, located in the extracellular domain, contain antigenic determinants capable of neutralizing monoclonal antibodies [23,24]. Research on the GP4 epitope across various PRRSV genotypes has demonstrated that the highly variable region of the GP4 protein in PRRSV-1 harbors a linear neutralizing epitope. The neutralizing antibodies elicited by this epitope exhibit specificity solely towards homologous strains [25]. A B-cell immunodominant epitope of GP4 (amino acids 50–67) was identified using synthetic overlapping peptides. This region was further confirmed to contain the core neutralizing epitope, thereby refining the understanding of antigenic epitope distribution within the GP4 protein [26].

## 3. Genetic Variation Analysis of the PRRSV *GP4* Gene

To assess the genetic variation of the PRRSV *GP4* gene, He et al. (2015) [27] analyzed the nucleotide and amino acid sequences of GP4 from 11 strains isolated between 2013 and 2014 using phylogenetic and sequence identity analyses. High amino acid identity (93.9–100%) was observed among these isolates. In comparison with the American classical strain VR-2332, the GP4 protein displayed 89.4% to 91.1% identity, while exhibiting 95.0% to 99.4% identity with Chinese HP-PRRSV strains. The lowest identity (55.9–72.6%) was noted with the European representative strain, the Lelystad virus.

The TJM vaccine strain was generated via 92 serial passages of the virus in Marc-145 cells (an African green monkey kidney epithelial cell line) [28]. Xue et al. (2012) [28] discovered that during this process, the F19 generation of the TJ strain exhibited mutations in the GP4 protein at several sites, mirroring those in HP-PRRSV strains, including V9 → L9, L15 → F15, and A32 → S32, among others. Additionally, the GXNN1310b and 10-10GX-1 strains displayed identical mutations at positions 3–5, with a change from “SSL” to “APF” [29].

An et al. (2011) [30] developed the JXA1-R vaccine strain through 80 serial passages of the highly pathogenic PRRSV JXA1 strain in Marc-145 cells. Compared to the JXA1 parental strain, the JXA1-R vaccine strain exhibited six amino acid mutations in the GP4 protein, which correspond to a mutation rate of 3.37%. During the development of the HuN4-F112 vaccine strain, obtained by 112 serial passages of the HP-PRRSV HuN4 strain in Marc-145 cells, five amino acid mutations were identified in the GP4 protein, resulting in a mutation rate of 2.81% [31].

Mutations in GP4 are distributed across multiple functional regions, including domains involved in CD163 receptor interaction and regions proximal to N-glycosylation sites. These mutations may influence interactions between viral particles and host cells, as well as glycosylation patterns, thereby potentially affecting infectivity and immune evasion [32]. Structural modifications induced by these mutations may impair interactions with host cell receptors, leading to changes in the core biological functions of GP4. In addition, disruption of viral particle assembly may occur, which could result in reduced virulence. Collectively, such changes may have substantial implications for infectivity and pathogenicity, consequently influencing vaccine efficacy and the dynamics of viral transmission [33].

Sun et al. (2013) [34] investigated the local Yanbian strain YB-0605 and identified pronounced sequence divergences in the N-terminal and central regions of the GP4 protein compared with European LV strains. These observations suggest that GP4 evolution in geographically isolated strains may display distinct regional characteristics, likely reflecting adaptive modifications driven by local environmental conditions and host–virus interactions.The key mutations identified in these vaccine and field strains are summarized in Table 1.

## 4. Interaction Between GP4 and Host Protein

CD163 is a member of the class I scavenger receptor family and is characterized as a 130 kDa transmembrane protein. The SRCR5 domain of CD163 is crucial for interaction with the GP4 protein [35]. Studies involving immunoprecipitation and molecular docking have confirmed the specific binding between CD163 and GP4, suggesting that small molecule drugs could potentially antagonize this interaction to inhibit viral infection [36]. The binding of GP4 to CD163 has been demonstrated to be a critical step in the initiation of viral infection in host cells [37]. During this process, GP4 anchors to the host cell surface through its interaction with CD163, thereby triggering signaling pathways that promote fusion between the viral envelope and the cell membrane. This fusion facilitates the entry of the viral genome into the host cell, effectively commencing the infection process [38].

The GP4 protein of PRRSV has been reported to interact with the host CD163 receptor and the cellular protein calpain 1; this interaction has been suggested to facilitate early viral uncoating, promotes the release of the viral genome into the cytoplasm, and initiates the subsequent viral genome replication process [39]. Upon the absence of the SRCR5 domain in porcine CD163, the interaction between PRRSV, and the cellular protein calpain 1 is abrogated, ultimately leading to the clearance of PRRSV by host cells [40]. During the canonical infection process of PRRSV, following PRRSV entry into host cells, porcine CD163 binds to PRRSV GP4 and concurrently mediates the recruitment of the cellular protein calpain 1 to the vicinity of PRRSV virions [41]. Calpain 1 has been shown to exert its protease activity to cleave and modify PRRSV structural proteins or associated cellular factors, which disrupts the structural stability of PRRSV virions and facilitates the release of the PRRSV genome from its capsid. This process establishes the prerequisite for subsequent PRRSV genomic replication [42,43].

Heat shock cognate 71 kDa protein (HSPA8) has been reported to interact with PRRSV GP4 via its C-terminal peptide-binding (PB) domain and to be involved in clathrin-dependent endocytosis during viral entry into cells [44]. Studies using chemical inhibitors and siRNA-mediated knockdown consistently showed that suppression of HSPA8 was associated with reduced PRRSV RNA levels, infectivity, and viral titers [45,46,47]. In addition, HSPA8–GP4 interaction was reported to induce conformational changes in PRRSV virions, potentially exposing additional receptor-binding sites and enhancing viral attachment to host cells [48].

**Figure 2:**
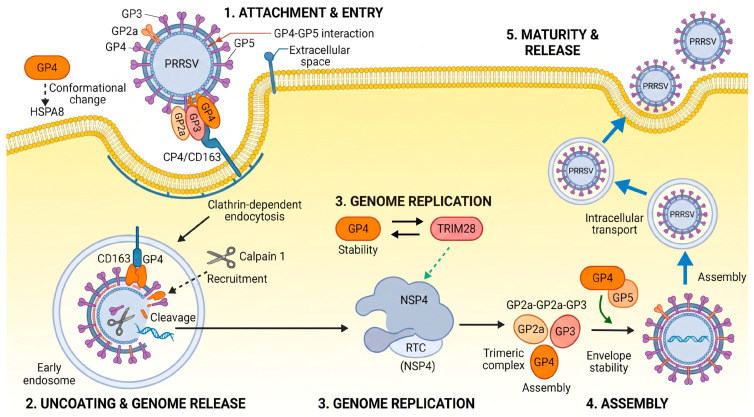
GP4 interacts with host and viral protein. The pattern was created with BioGDP [19].

In addition, interactions between PRRSV GP4 and the cellular protein HSPA8 have been associated with modulation of viral attachment to cells [49]. The schematic summary of these processes were shown in Figure 2.

## 5. Interactions Between GP4 and Other PRRSV Proteins

Wissink et al. (2005) [50] investigated the characteristics and functions of PRRSV non-structural proteins (NSPs) and demonstrated that these NSPs contribute significantly to viral replication. During PRRSV replication, PRRSV GP4 may exhibit potential interactions with NSPs; for instance, it may engage in indirect interactions with NSP4, thereby promoting PRRSV genomic replication by regulating the activity of the viral replication complex (RTC) [51]. The GP4 protein of PRRSV can form a heteromultimeric complex with the minor structural proteins GP2a and GP3, and this complex plays a crucial role in PRRSV virion assembly and host cell infection. Furthermore, the potential interaction between PRRSV GP4 and GP5 may contribute to the stable maintenance of the PRRSV envelope structure [52]. Specifically, the specific interaction between PRRSV GP4 and GP2a enables this subcomplex to serve as a viral attachment entity, which mediates the engagement of PRRSV with the porcine CD163 receptor during the virus entry into susceptible host cells [53,54].

The PRRSV GP4 protein has been implicated in viral replication through its involvement in the intracellular transport of viral components to the host cell surface, a process associated with efficient virion release. In addition, during viral assembly, the GP2a–GP3–GP4 trimeric envelope protein complex has been reported to constitute a key component contributing to efficient PRRSV assembly [55,56].

GP4 modulates replication efficiency through interactions with cellular proteins such as TRIM28. During assembly, GP4 forms a heteromultimeric complex with GP2a and GP3, which is essential for virion assembly, and interacts with GP5 to maintain envelope stability. During release, GP4 facilitates intracellular transport of viral components to the cell surface [22,57,58]. The schematic summary of these processes is shown in Figure 2.

## 6. Relationship Between GP4 and Host Immune Response

The GP4 protein of PRRSV has been demonstrated to be immunogenic and capable of inducing the production of PRRSV-specific antibodies in the host, thereby contributing to humoral immune responses against PRRSV infection [16]. Through the application of synthetic peptide technology, the B-cell epitope of the PRRSV GP4 protein was screened, and several dominant peptide segments were identified—most notably the fourth segment, with amino acid residues 51–65 or 59–67 serving as the core region of the neutralizing epitope [59]. Antibodies targeting this peptide were detectable at 3–5 weeks post-immunization, indicating that these GP4-derived epitopes effectively elicit PRRSV-specific antibody production and robust humoral immune responses in the host [60].

Chen et al. (2025) [61] identified key motifs mediating the interaction between residues Q48 and I5 of the PRRSV GP4 protein and the monoclonal antibody mAb-5F2. It was subsequently demonstrated that mAb-5F2 spatially interferes with the interaction between PRRSV GP4 and the porcine receptor CD163, resulting in a 78–85% reduction in viral entry efficiency at a concentration of 50 µg/mL [62,63]. These findings not only confirm the presence of critical neutralizing epitopes on the GP4 protein but also demonstrate that PRRSV infection can be effectively inhibited by antibodies targeting these epitopes, thereby underscoring the essential role of GP4 in the induction of protective humoral immune responses [64,65,66].

During PRRSV replication in host cells, viral antigens are expressed on the surface of infected cells and presented via major histocompatibility complex class I (MHC-I) molecules. As a result, infected cells are recognized as non-self and targeted by immune effector mechanisms [67]. T-cell immunodominant peptides derived from the GP4 protein have been identified and mapped to amino acid residues 41–58, 86–103, and 140–157 within the PRRSV GP4 sequence. These regions are therefore capable of being recognized by T cells, leading to the activation of cellular immune responses [68,69]. The cytotoxic effects mediated by T lymphocytes can directly kill virus-infected cells, preventing the spread of the virus between cells [70,71,72]. Consequently, the cell-mediated immune response induced by the GP4 protein plays a significant role in limiting viral replication and controlling infection [73].

GP4 has been demonstrated to induce a neutralizing antibody response, with GP4-specific antibodies detected in approximately 65% of PRRSV-2-positive sera, suggesting that GP4 is immunogenic in a substantial proportion of infected pigs [74,75].

## 7. Relationship Between GP4 and Virulence

Compared with the PRRSV VR-2332 strain, the conformational epitope of the GP4 protein in the Yan-Bian PRRSV strain YB-0605 exhibited significant structural changes within the amino acid residues 30–40 region, and the immunodominance of the epitope at residue 50 was enhanced—a phenomenon that may be associated with viral virulence and immune escape [34]. Mutations at multiple amino acid sites in the GP4 protein of the Guangxi PRRSV isolate are consistent with those of HP-PRRSV, and aa residue 83 of this isolate is serine (Ser83), indicating that this site or its flanking regions are closely correlated with viral virulence [27].

Changes in the receptor-binding capacity of GP4 or its synergistic efficiency with other viral proteins may impair PRRSV entry into host cells, thereby reducing viral replication and transmissibility, and ultimately modulating virulence [76,77]. PRRSV GP4 is also implicated in the assembly and release phases of PRRSV virions, and the integrity of its functional activity modulates the formation and release yield of PRRSV virions. When PRRSV assembly or release processes are abrogated, viral transmissibility is constrained, with concomitant reduction in virulence [78].

VR-2332, a representative strain of PRRSV-2, was serially passaged in vitro in Marc-145 cells to generate the RespPRRS MLV vaccine strain [52]. During this passage process, no amino acid mutations were identified in the GP4 protein, and the mutation rate was 0% [79].

Chimeric strains with different regions of the GP4 protein replaced were constructed using reverse genetics [80]. The CHsx1401-SPJX strain, which carries the complete JXwn06 structural protein region, was used as the control. Twenty-eight-day-old SPF pigs were randomly assigned to groups and intranasally inoculated with experimental viruses or DMEM. Except for the CHsx1401-SPJX group, viremia was detected from day 3 post-inoculation in all groups and persisted until day 14. Significant viremia was observed in the DMEM and CHsx1401 groups between days 3 and 5, and clearance in the CHsx1401 group occurred only by day 14. In contrast, only low-level viremia was detected in the chimeric virus–immunized groups and was rapidly cleared between days 5 and 7. Viremia was undetectable from day 3 in the CHsx1401-SPJX and CHsx1401-GP5MJX groups [81,82]. These findings suggest that the GP4 protein indirectly modulates PRRSV virulence by influencing viral entry, participating in virion assembly and release, and regulating host immune response efficiency, ultimately leading to attenuated pathogenicity [1,83].

## 8. Application of GP4 Protein in Vaccine Development

The GP4 protein has been considered an ideal target for subunit vaccine development due to its essential role in viral infection and its strong immunogenicity [84].

Although GP4 is naturally glycosylated in eukaryotic cells, prokaryotic expression systems lack this post-translational modification. Nevertheless, recombinant GP4 expressed in *E. coli* has been shown to retain immunoreactivity with PRRSV-positive sera, suggesting that linear epitopes, rather than conformational glycosylation-dependent epitopes, are sufficient for antibody detection and certain vaccine applications [85].

Numerous studies have employed prokaryotic expression systems, such as the SUMO system, pET32a vector, and *E. coli* system, to express or co-express the GP4 recombinant protein. Following purification, these studies confirmed that the protein could induce a specific immune response, bind specifically to PRRSV-positive sera, and be used to produce high-titer polyclonal antibodies, thereby providing a solid foundation for the development of GP4 subunit vaccines [86,87,88].

Jing et al. (2023) [89] constructed a recombinant lymphocytic choriomeningitis virus (LCMV) reverse genetics system carrying the GP4 gene. They found that after several passages, the recombinant virus exhibited enhanced proliferative ability and could express the GP4 protein, offering a new vector direction for PRRSV vaccine development.

Codon-optimized GP4 lacking the transmembrane region was expressed in Arabidopsis thaliana [90]. Following oral administration of transgenic plant material to pigs, significant humoral and cellular immune responses were induced, and lung injury and viral load were reduced upon challenge [91,92], suggesting that plant-derived GP4 may serve as an effective subunit vaccine component.

Guo et al. (2022) [93] truncated the GP4 protein for monoclonal antibody production and constructed a stable Marc-145 cell line expressing the ORF4 gene. Through trans-complementation rescue, they obtained an ORF4 replication-defective PRRSV, providing candidate strains for a novel replication-defective vaccine.

## 9. Application of GP4 Protein in Antibody Detection

Prokaryotic expression systems have been widely used to produce recombinant GP4 protein. After mouse immunization, high-titer polyclonal antibodies (up to 1:12,800) or GP4-specific monoclonal antibodies (e.g., 5F12 and four IgG isotype–positive clones) were successfully generated. These antibodies exhibited high specificity and stability and were able to specifically recognize GP4 protein or PRRSV. Therefore, they provided essential antibody resources for the development of PRRSV diagnostic methods and reagents [85,94,95,96,97,98].

Mice were immunized with prokaryotically expressed GP4 protein derived from the PRRSV HUN4 strain, and GP4-specific monoclonal antibodies were generated. The peptide epitopes recognized by these antibodies were subsequently identified using a phage-displayed random peptide library [99,100,101]. These findings provided a foundation for PRRSV detection and epitope-based vaccine research design.

Collectively, these findings indicate that bacterially expressed GP4 is well suited for generating high-specificity antibody reagents and for defining antigenic determinants relevant to PRRSV diagnostics and antigen design.

## 10. Application of GP4 Protein in Therapeutic Antibodies

The conserved neutralizing epitope of the GP4 protein provides a critical target for the development of therapeutic monoclonal antibodies [61]. Porcine-derived monoclonal antibodies, owing to their high identity with the host and low immunogenicity, have shown significant potential in the treatment of PRRSV infections [102]. Recent studies reported a porcine monoclonal antibody targeting the conserved GP4 epitope, presenting a breakthrough approach for PRRS treatment. This study developed porcine monoclonal antibodies targeting the conserved neutralizing epitope of GP4. In vitro experiments confirmed that the antibody specifically binds to different PRRSV genotypes and blocks the interaction between GP4 and the host CD163 receptor through steric hindrance. The antibody demonstrated broad-spectrum neutralizing activity, significantly outperforming traditional mouse-derived monoclonal antibodies.

Challenge experiments further validated the protective effects of the antibody. After administration to 28-day-old SPF pigs, followed by intranasal exposure to a highly virulent PRRSV strain, no significant clinical symptoms were observed in the pigs. The duration of viremia was reduced by more than 50%, lung viral load was significantly lower, and lung tissue damage scores were reduced by 60% compared to the control group, no antibody-dependent enhancement (ADE) was observed [103].

These results indicate that porcine monoclonal antibodies targeting the conserved GP4 epitope can effectively control PRRSV infection by inducing broad-spectrum neutralizing responses, offering a novel strategy for treating emergency PRRS outbreaks in pig populations in vivo [104].

A recent study employed phage display technology to screen for GP4-specific nanobodies, identifying Nb6, Nb31, and Nb85. It was demonstrated by functional assays that these nanobodies effectively inhibit PRRSV infection via blocking viral attachment to host cells and subsequent internalization in vivo. These findings offer novel candidate formulations for PRRSV antiviral therapy and broaden the potential applications of GP4 protein in the development of therapeutic biopharmaceuticals [105].

## 11. Future Directions

PRRSV represents a significant threat to the swine industry, with its GP4 protein playing a pivotal role in viral entry and immune evasion. Consequently, GP4 has become a central target for control and prevention research. Future investigations should prioritize three key areas: structural-functional analysis, technological innovation and translation, and integrated control strategies. These efforts are essential for providing both theoretical support and technological solutions for the precise prevention and control of PRRS.

In structural studies, cryo-electron microscopy (cryo-EM) and AI modeling techniques should be utilized to determine the full-length three-dimensional structure of the GP4 protein [106]. This will provide insights into the biological functions of its glycosylation modifications, reveal the assembly mechanism of the GP4 complex with GP2a and GP3, and confirm the role of critical amino acid residues. Furthermore, the interaction sites and pathways between GP4 and the host receptor CD163 require further exploration. A comprehensive understanding of the regulatory mechanisms of viral immune evasion will establish a foundation for future research. Similar to PRRSV GP4, the GP2-GP3-GP4 complex of equine arteritis virus (EAV) has been shown to determine cell tropism and receptor binding, suggesting that the GP4-mediated entry mechanism may be conserved among *Arteriviruses* [18].

In the context of vaccine development, multi-epitope vaccine candidates can be rationally designed based on conserved regions of the GP4 protein. The SpyCatcher/SpyTag system may be utilized to incorporate immunodominant epitopes from GP3, GP5, and other viral proteins to enhance overall immunogenicity [107]. Meanwhile, emerging vaccine platforms, including self-amplifying RNA (saRNA) vaccines and nanoparticle-based vaccines, warrant further exploration. These technologies offer advantages such as low-dose administration and high antigen expression and may enable efficient presentation of GP4 antigens while synergistically activating both humoral and cellular immune responses. In influenza virus, hemagglutinin (HA) receptor-binding domain mutations and glycosylation changes drive immune escape, highlighting the importance of targeting conserved epitopes in vaccine design [108].

Regarding diagnostic applications, high-affinity monoclonal antibodies targeting conserved GP4 epitopes should be developed to improve the sensitivity and specificity of ELISA kits and colloidal gold immunochromatographic assays. Such improvements would enhance the detection of variant strains and early-stage infections. Furthermore, the establishment of a dual diagnostic strategy combining antigen detection with nucleic acid confirmation is recommended. By integrating colloidal gold assays with RT-qPCR, a standardized workflow from preliminary screening to confirmatory diagnosis can be achieved to accommodate diverse clinical and field scenarios.

## 12. Conclusions

An integrated, multi-technology framework centered on the GP4 protein, complemented by continuous surveillance of conserved GP4 sequences and epidemiological monitoring of circulating strains, may contribute to more effective PRRS prevention and control in the future.

## Figures and Tables

**Table 1:** Summary of amino acid mutations in the PRRSV GP4 protein.

Strain/Vaccine	Parental Strain	GP4 Mutations	Mutation Rate
TJM vaccine strain	HP-PRRSV TJ strain	V9 → L9, L15 → F15, A32 → S32, among others (detected at passage 19)	—
GXNN1310b/10-10GX-1	Guangxi isolates	“SSL” → “APF” at aa positions 3–5	—
JXA1-R vaccine strain	HP-PRRSV JXA1 strain	6 amino acid mutations	3.37%
HuN4-F112 vaccine strain	HP-PRRSV HuN4 strain	5 amino acid mutations	2.81%
YB-0605	Yanbian isolate	Epitope morphology at aa 30–40 significantly altered; immunodominance at aa 50 enhanced	—
Guangxi isolate	Guangxi isolate	Serine at aa position 83 (Ser83)	—

## Data Availability

All datasets are available in the main manuscript. The dataset sup porting the conclusions of this article is included within the article.

## References

[1] Lunney J.K., Fang Y., Ladinig A., Chen N., Li Y., Rowland B., Renukaradhya G.J. (2016). Porcine Reproductive and Respiratory Syndrome Virus (PRRSV): Pathogenesis and Interaction with the Immune System. Annu. Rev. Anim. Biosci..

[2] Nathues H., Alarcon P., Rushton J., Jolie R., Fiebig K., Jimenez M., Geurts V., Nathues C. (2017). Cost of porcine reproductive and respiratory syndrome virus at individual farm level—An economic disease model. Prev. Vet. Med..

[3] Bilodeau R., Dea S., Sauvageau R.A., Martineau G.P. (1991). ‘Porcine reproductive and respiratory syndrome’ in Quebec. Vet. Rec..

[4] Shang Y., Wang G., Yin S., Tian H., Du P., Wu J., Chen Y., Yang S., Jin Y., Zhang K. (2013). Pathogenic characteristics of three genotype II porcine reproductive and respiratory syndrome viruses isolated from China. Virol. J..

[5] Christianson W.T., Choi C.S., Collins J.E., Molitor T.W., Morrison R.B., Joo H.S. (1993). Pathogenesis of porcine reproductive and respiratory syndrome virus infection in mid-gestation sows and fetuses. Can. J. Vet. Res..

[6] Wang H. (2021). Evolutionary trend of PRRSV in recent years in China and its impact on prevention and control strategy. Chin. J. Anim. Infect. Dis..

[7] Benfield D.A., Nelson E., Collins J.E., Harris L., Goyal S.M., Robison D., Christianson W.T., Morrison R.B., Gorcyca D., Chladek D. (1992). Characterization of swine infertility and respiratory syndrome (SIRS) virus (isolate ATCC VR-2332). J. Vet. Diagn. Investig..

[8] Darwich L., Gimeno M., Sibila M., Diaz I., de la Torre E., Dotti S., Kuzemtseva L., Martin M., Pujols J., Mateu E. (2011). Genetic and immunobiological diversities of porcine reproductive and respiratory syndrome genotype I strains. Vet. Microbiol..

[9] Montaner-Tarbes S., Del Portillo H.A., Montoya M., Fraile L. (2019). Key Gaps in the Knowledge of the Porcine Respiratory Reproductive Syndrome Virus (PRRSV). Front. Vet. Sci..

[10] Meulenberg J.J., van Nieuwstadt A.P., van Essen-Zandbergen A., Langeveld J.P. (1997). Posttranslational processing and identification of a neutralization domain of the GP4 protein encoded by ORF4 of Lelystad virus. J. Virol..

[11] Meulenberg J.J., de Meijer E.J., Moormann R.J. (1993). Subgenomic RNAs of Lelystad virus contain a conserved leader-body junction sequence. J. Gen. Virol..

[12] Kappes M.A., Faaberg K.S. (2015). PRRSV structure, replication and recombination: Origin of phenotype and genotype diversity. Virology.

[13] Yim-Im W., Anderson T.K., Paploski I.A.D., VanderWaal K., Gauger P., Krueger K., Shi M., Main R., Zhang J. (2023). Refining PRRSV-2 genetic classification based on global ORF5 sequences and investigation of their geographic distributions and temporal changes. Microbiol. Spectr..

[14] Gao Z.Q., Guo X., Yang H.C. (2004). Genomic characterization of two Chinese isolates of porcine respiratory and reproductive syndrome virus. Arch. Virol..

[15] Snijder E.J., Kikkert M., Fang Y. (2013). Arterivirus molecular biology and pathogenesis. J. Gen. Virol..

[16] Vanhee M., Costers S., Van Breedam W., Geldhof M.F., Van Doorsselaere J., Nauwynck H.J. (2010). A variable region in GP4 of European-type porcine reproductive and respiratory syndrome virus induces neutralizing antibodies against homologous but not heterologous virus strains. Viral Immunol..

[17] Li D., Zhu L., Cui C., Wu Z., Qing P., Zhou Q., Gao P., Zhang Y., Zhou L., Ge X. (2025). The role of major and minor structural proteins of porcine reproductive and respiratory syndrome virus in induction of protective immunity. Front. Microbiol..

[18] Veit M., Matczuk A.K., Sinhadri B.C., Krause E., Thaa B. (2014). Membrane proteins of arterivirus particles: Structure, topology, processing and function. Virus Res..

[19] Jiang S., Li H., Zhang L., Mu W., Zhang Y., Chen T., Wu J., Tang H., Zheng S., Liu Y. (2025). Generic Diagramming Platform (GDP): A comprehensive database of high-quality biomedical graphics. Nucleic Acids Res..

[20] Wieringa R., de Vries A.A., Raamsman M.J., Rottier P.J. (2002). Characterization of two new structural glycoproteins, GP(3) and GP(4), of equine arteritis virus. J. Virol..

[21] Du Y., Pattnaik A.K., Song C., Yoo D., Li G. (2012). Glycosyl-phosphatidylinositol (GPI)-anchored membrane association of the porcine reproductive and respiratory syndrome virus GP4 glycoprotein and its co-localization with CD163 in lipid rafts. Virology.

[22] Dea S., Gagnon C.A., Mardassi H., Pirzadeh B., Rogan D. (2000). Current knowledge on the structural proteins of porcine reproductive and respiratory syndrome (PRRS) virus: Comparison of the North American and European isolates. Arch. Virol..

[23] Welch S.K., Jolie R., Pearce D.S., Koertje W.D., Fuog E., Shields S.L., Yoo D., Calvert J.G. (2004). Construction and evaluation of genetically engineered replication-defective porcine reproductive and respiratory syndrome virus vaccine candidates. Vet. Immunol. Immunopathol..

[24] Li L. (2014). Identification of a B-Cell Epitope in Thenucleocapsid Protein of Highly Pathogenic Porcinereproductive and Respiratory Syndrome virusHuN4 Strain. Master’s Thesis.

[25] Das P.B., Dinh P.X., Ansari I.H., de Lima M., Osorio F.A., Pattnaik A.K. (2010). The minor envelope glycoproteins GP2a and GP4 of porcine reproductive and respiratory syndrome virus interact with the receptor CD163. J. Virol..

[26] Wang A., Chen B., Liu H., Zhou J., Chen Y., Liu Y., Qi Y., Zhang G. (2019). Screening B Cell Epitopes of GP4 Protein from Porcine Reproductive and Respiratory Syndrome Virus. Acta Agric. Boreali Occident. Sin..

[27] He W., Wei Y., Huang J., Hong S., Lin S., Yao J., Chen Y., Huang W., Wei Z. (2015). Genetic evolution of GP2, GP3 and GP4 proteins of porcine reproductive and respiratory syndrome virus origin of Guangxi. Chin. J. Anim. Infect. Dis..

[28] Xue L., Li Z., Xia M., Li X., Wang F., Wang W., Zhang X., Wu H. (2012). Mutations in the genome of the highly pathogenic porcine reproductive and respiratory syndrome virus potentially related to attenuation. Vet. Microbiol..

[29] Chen J.Z., Peng J.M., Bai Y., Wang Q., Liu Y.M., Zhang Q.Y., Chang D., Zhang W.C., Zhao H.Y., Ye C. (2015). Characterization of two novel porcine reproductive and respiratory syndrome virus isolates with deletions in the GP2 gene. Vet. Microbiol..

[30] An T.Q., Tian Z.J., Zhou Y.J., Xiao Y., Peng J.M., Chen J., Jiang Y.F., Hao X.F., Tong G.Z. (2011). Comparative genomic analysis of five pairs of virulent parental/attenuated vaccine strains of PRRSV. Vet. Microbiol..

[31] Wu Q., Li Z., Zhang G., Niu J., Zeng X., Sun B., Ma J. (2017). Genetic diversity and phylogenetic analysis of porcine reproductive and respiratory syndrome virus in southern China from 2007 to 2014. J. Vet. Sci..

[32] Fu L., Yang H., Guo X., Chen Y. Prokaryotic expression of double epitope tandem repeats of PRRSV GP4 and GP5 and their immune effects induced in mice. Proceedings of the 6th National Congress and 11th Academic Seminar of Animal Infectious Diseases Branch of China Animal Husbandry and Veterinary Society.

[33] Yu X., Chen N., Deng X., Cao Z., Han W., Hu D., Wu J., Zhang S., Wang B., Gu X. (2013). Genomic sequencing reveals mutations potentially related to the overattenuation of a highly pathogenic porcine reproductive and respiratory syndrome virus. Clin. Vaccine Immunol. CVI.

[34] Sun C., Cai J., Guan L., Lou A. (2013). Analysis of GP4 structural gene of porcine reproductive and respiratory syndrome virus Yanbian local strain. Jiangsu Agric. Sci..

[35] Burkard C., Opriessnig T., Mileham A.J., Stadejek T., Ait-Ali T., Lillico S.G., Whitelaw C.B.A., Archibald A.L. (2018). Pigs Lacking the Scavenger Receptor Cysteine-Rich Domain 5 of CD163 Are Resistant to Porcine Reproductive and Respiratory Syndrome Virus 1 Infection. J. Virol..

[36] Huang C., Bernard D., Zhu J., Dash R.C., Chu A., Knupp A., Hakey A., Hadden M.K., Garmendia A., Tang Y. (2020). Small molecules block the interaction between porcine reproductive and respiratory syndrome virus and CD163 receptor and the infection of pig cells. Virol. J..

[37] Zhu J., He X., Bernard D., Shen J., Su Y., Wolek A., Issacs B., Mishra N., Tian X., Garmendia A. (2023). Identification of New Compounds against PRRSV Infection by Directly Targeting CD163. J. Virol..

[38] Rowland R.R.R., Brandariz-Nuñez A. (2024). Role of CD163 in PRRSV infection. Virology.

[39] Cai H., Zhang H., Cheng H., Liu M., Wen S., Ren J. (2023). Progress in PRRSV Infection and Adaptive Immune Response Mechanisms. Viruses.

[40] Yu P., Wei R., Dong W., Zhu Z., Zhang X., Chen Y., Liu X., Guo C. (2019). CD163(ΔSRCR5) MARC-145 Cells Resist PRRSV-2 Infection via Inhibiting Virus Uncoating, Which Requires the Interaction of CD163 with Calpain 1. Front. Microbiol..

[41] Amona F.M., Pang Y., Gong X., Wang Y., Fang X., Zhang C., Chen X. (2024). Mechanism of PRRSV infection and antiviral role of polyphenols. Virulence.

[42] Burkard C., Lillico S.G., Reid E., Jackson B., Mileham A.J., Ait-Ali T., Whitelaw C.B., Archibald A.L. (2017). Precision engineering for PRRSV resistance in pigs: Macrophages from genome edited pigs lacking CD163 SRCR5 domain are fully resistant to both PRRSV genotypes while maintaining biological function. PLoS Pathog..

[43] Clilverd H., Li Y., Martín-Valls G., Aguirre L., Martín M., Cortey M., Mateu E. (2024). Selection of viral variants with enhanced transmission and reduced neutralization susceptibility alongside lateral introductions may explain the persistence of porcine reproductive and respiratory syndrome virus in vaccinated breeding herds. Virus Evol..

[44] Wang L., Li R., Geng R., Zhang L., Chen X.X., Qiao S., Zhang G. (2022). Heat Shock Protein Member 8 (HSPA8) Is Involved in Porcine Reproductive and Respiratory Syndrome Virus Attachment and Internalization. Microbiol. Spectr..

[45] Ling X., Cao Z., Sun P., Zhang H., Sun Y., Zhong J., Yin W., Fan K., Zheng X., Li H. (2023). Target Discovery of Matrine against PRRSV in Marc-145 Cells via Activity-Based Protein Profiling. Int. J. Mol. Sci..

[46] Zhang C., Wang Y., Wu G., Sun N., Bai H., Li X., Han S., Zhou H., Qi R., Zhang J. (2024). RPL35A promotes the progression of cholangiocarcinoma by mediating HSPA8 ubiquitination. Biol. Direct.

[47] Han S., Oh D., Vanderheijden N., Xie J., Balmelle N., Tignon M., Nauwynck H.J. (2025). Monoclonal Antibodies Targeting Porcine Macrophages Are Able to Inhibit the Cell Entry of Macrophage-Tropic Viruses (PRRSV and ASFV). Viruses.

[48] Pei Y., Lin C., Li H., Feng Z. (2023). Genetic background influences pig responses to porcine reproductive and respiratory syndrome virus. Front. Vet. Sci..

[49] Sun Z., Chen Z., Lawson S.R., Fang Y. (2010). The cysteine protease domain of porcine reproductive and respiratory syndrome virus nonstructural protein 2 possesses deubiquitinating and interferon antagonism functions. J. Virol..

[50] Wissink E.H., Kroese M.V., van Wijk H.A., Rijsewijk F.A., Meulenberg J.J., Rottier P.J. (2005). Envelope protein requirements for the assembly of infectious virions of porcine reproductive and respiratory syndrome virus. J. Virol..

[51] You S.S. (2025). Establishment of PRRSV MultiplexDigital PCR Method and Preparation ofGP2/GP3/GP4 Monoclonal Antibodies. Master’s Thesis.

[52] Meulenberg J.J., van Nieuwstadt A.P., van Essen-Zandbergen A., Bos-de Ruijter J.N., Langeveld J.P., Meloen R.H. (1998). Localization and fine mapping of antigenic sites on the nucleocapsid protein N of porcine reproductive and respiratory syndrome virus with monoclonal antibodies. Virology.

[53] Chen W., Cui J., Wang J., Sun Y., Ji C., Song R., Zeng Y., Pan H., Sheng J., Zhang G. (2018). Phages bearing specific peptides with affinity for porcine reproductive and respiratory syndrome virus GP4 protein prevent cell penetration of the virus. Vet. Microbiol..

[54] Ouyang Y., Zhou Y., Fang L., Xiao S. (2022). Analysis on the Prevalence and Genetic Characteristics of PRRSV in Pig Concentrated Breeding Areas of China. Prog. Vet. Med..

[55] An T.Q., Tian Z.J., Xiao Y., Li R., Peng J.M., Wei T.C., Zhang Y., Zhou Y.J., Tong G.Z. (2010). Origin of highly pathogenic porcine reproductive and respiratory syndrome virus, China. Emerg. Infect. Dis..

[56] Han J., Liu G., Wang Y., Faaberg K.S. (2007). Identification of nonessential regions of the nsp2 replicase protein of porcine reproductive and respiratory syndrome virus strain VR-2332 for replication in cell culture. J. Virol..

[57] Delputte P.L., Nauwynck H.J. (2004). Porcine arterivirus infection of alveolar macrophages is mediated by sialic acid on the virus. J. Virol..

[58] Lv C., Yang Z., Lan X., Liang F., Kong W., Wang R., Zhao M. (2025). Research Progress on the GP3 Protein of Porcine Reproductive and Respiratory Syndrome Virus. Animals.

[59] Du T., Nan Y., Xiao S., Zhao Q., Zhou E.M. (2017). Antiviral Strategies against PRRSV Infection. Trends Microbiol..

[60] Zhang Q., Yoo D. (2015). PRRS virus receptors and their role for pathogenesis. Vet. Microbiol..

[61] Chen X., Zhao J., Ji P., Li X., Niu H., Jiao D., Zhang L., Zhu Q., Liu X., Hiscox J.A. (2025). A Porcine Monoclonal Antibody Targeting a Conserved GP4 Epitope Protects against In Vivo Infection via the Induction of Broad-Spectrum PRRSV Neutralization. Adv. Sci..

[62] de Brito R.C.F., Holtham K., Roser J., Saunders J.E., Wezel Y., Henderson S., Mauch T., Sanz-Bernardo B., Frossard J.P., Bernard M. (2023). An attenuated herpesvirus vectored vaccine candidate induces T-cell responses against highly conserved porcine reproductive and respiratory syndrome virus M and NSP5 proteins that are unable to control infection. Front. Immunol..

[63] Cheng Y., Wu M., Xiao L., Zhang M., Huang B., Cong F., Yi L. (2023). Identificationof a novel linear epitope on the porcine reproductive and respiratory syndrome virus nucleocapsid protein, as recognized by a specific monoclonal antibody. Front. Immunol..

[64] Huang Y., Zhang B., Zhang L., Mayer B.T., Martin T., Hahn W., Hyrien O., Gelderblom H.C. (2025). Dose finding in early-phase human immunodeficiency virus type 1 prevention monoclonal antibody clinical trials. Clin. Trials.

[65] Vargas-Bermudez D.S., Mogollon J.D., Jaime J. (2025). Effects of Primary Viruses (PCV2, PPV1, and PRRSV) Involved in Porcine Reproductive Failure as Mono- and Coinfections with Each Other and with Emerging Viruses (PCV3 and nPPVs). Viruses.

[66] Gao J.C., Xiong J.Y., Ye C., Chang X.B., Guo J.C., Jiang C.G., Zhang G.H., Tian Z.J., Cai X.H., Tong G.Z. (2017). Genotypic and geographical distribution of porcine reproductive and respiratory syndrome viruses in mainland China in 1996–2016. Vet. Microbiol..

[67] Robinson S.R., Rahe M.C., Gray D.K., Martins K.V., Murtaugh M.P. (2018). Porcine reproductive and respiratory syndrome virus neutralizing antibodies provide in vivo cross-protection to PRRSV1 and PRRSV2 viral challenge. Virus Res..

[68] Chen B., Liu H.L., Li J.G., Li Z.H., Zhang G.P. Screening of T Cell Epitopes of GP4 Protein of Porcine Reproductive and Respiratory Syndrome Virus. Proceedings of the 13th National Immunology Academic Conference.

[69] Sha H., Zhang H., Chen Y., Huang L., Zhao M., Wang N. (2022). Research Progress on the NSP9 Protein of Porcine Reproductive and Respiratory Syndrome Virus. Front. Vet. Sci..

[70] Tian K., Yu X., Zhao T., Feng Y., Cao Z., Wang C., Hu Y., Chen X., Hu D., Tian X. (2007). Emergence of fatal PRRSV variants: Unparalleled outbreaks of atypical PRRS in China and molecular dissection of the unique hallmark. PLoS ONE.

[71] Li J., Murtaugh M.P. (2012). Dissociation of porcine reproductive and respiratory syndrome virus neutralization from antibodies specific to major envelope protein surface epitopes. Virology.

[72] Oleksiewicz M.B., Bøtner A., Normann P. (2002). Porcine B-cells recognize epitopes that are conserved between the structural proteins of American- and European-type porcine reproductive and respiratory syndrome virus. J. Gen. Virol..

[73] Nan Y., Wu C., Gu G., Sun W., Zhang Y.J., Zhou E.M. (2017). Improved Vaccine against PRRSV: Current Progress and Future Perspective. Front. Microbiol..

[74] Liu MengYing L.M. (2019). Preparation of a Monoclonal Antibody with Neutralizing Activity Against North American PRRSV GP4 and Identification of Its Epitope. Master’s Thesis.

[75] Music N., Gagnon C.A. (2010). The role of porcine reproductive and respiratory syndrome (PRRS) virus structural and non-structural proteins in virus pathogenesis. Anim. Health Res. Rev..

[76] Castillo-Pérez J., Martínez-Lobo F.J., Frómeta R., Castro J.M., Simarro I., Prieto C. (2024). Linear epitopes of PRRSV-1 envelope proteins ectodomains are not correlated with broad neutralization. Porc. Health Manag..

[77] Wang X., Wang Z., Xu H., Biao X., Yang Z. (2016). A single amino acid substitution alter antigenicity of Glycosylated protein 4 of HP-PRRSV. Virol. J..

[78] Li J.H., Zhang W., Cheng L.L., Pan X.M., Tang H.F., Zhou T., Qing Y.M., Hu Y.K., Liu Q.D., Xiao Y. (2023). Virulence evaluation of porcine reproductive and respiratory syndrome virus NADC30-like strain. China Anim. Health.

[79] Dea S., Gagnon C.A., Mardassi H., Milane G. (1996). Antigenic variability among North American and European strains of porcine reproductive and respiratory syndrome virus as defined by monoclonal antibodies to the matrix protein. J. Clin. Microbiol..

[80] Sacramento C.Q., Bott R., Huang Q., Eaton B., Postnikova E., Sabir A.J., Argade M.D., Ratia K., Anantpadma M., Carlier P.R. (2025). Recombinant Pichinde reporter virus as a safe and suitable surrogate for high-throughput antiviral screening against highly pathogenic arenaviruses. Antivir. Res..

[81] Charerntantanakul W. (2009). Adjuvants for porcine reproductive and respiratory syndrome virus vaccines. Vet. Immunol. Immunopathol..

[82] Sánchez-Matamoros A., Camprodon A., Maldonado J., Pedrazuela R., Miranda J. (2019). Safety and long-lasting immunity of the combined administration of a modified-live virus vaccine against porcine reproductive and respiratory syndrome virus 1 and an inactivated vaccine against porcine parvovirus and Erysipelothrix rhusiopathiae in breeding pigs. Porc. Health Manag..

[83] Guo J., Li C., Lu H., Wang B., Zhang L., Ding J., Jiao X., Li Q., Zhu S., Wang A. (2024). Reverse genetics construction and pathogenicity of a novel recombinant NADC30-like PRRSV isolated in China. Front. Vet. Sci..

[84] Khan M. (2024). Development of PRRSV mRNA Vaccine and Its Immunoprotection Evaluation. Ph.D. Thesis.

[85] Das P.B., Vu H.L., Dinh P.X., Cooney J.L., Kwon B., Osorio F.A., Pattnaik A.K. (2011). Glycosylation of minor envelope glycoproteins of porcine reproductive and respiratory syndrome virus in infectious virus recovery, receptor interaction, and immune response. Virology.

[86] Li H., Zhang W., Wang W., Qiao Y., Xu M., Liu Z., Gu X., Wu A., Ma Z., Chen C. (2025). PRRSV GP4 subunit vaccine combined with adenovirus heterologous prime-boost immunization strategy induced a significant immune response in mice. BMC Vet. Res..

[87] Che Z., Shi X., Zhang G., Kou L., Zhou J., Qi Y., Wang A. (2017). Prokaryotic expression and purification of GP4 protein of porcinereproductive and respiratory syndrome virus BJ-4 strain. J. Northwest A F Univ..

[88] Ning H., Peng N., Chen Y., Li X., Fan J., Dong W. (2024). Prokaryotic Expression of PRRSV GP2-GP4 Protein and Preparation of Polyclonal Antibody. Mod. Anim. Husb. Sci. Technol..

[89] Jing H., Li H., Wang J., Cao S., Wang H., Zhang Y., Dong W., Bao W. (2023). Establishment and Identification of A Recombinant LCMV Expressing PRRSV GP2a-GP4. Prog. Vet. Med..

[90] An C.H., Nazki S., Park S.C., Jeong Y.J., Lee J.H., Park S.J., Khatun A., Kim W.I., Park Y.I., Jeong J.C. (2018). Plant synthetic GP4 and GP5 proteins from porcine reproductive and respiratory syndrome virus elicit immune responses in pigs. Planta.

[91] Han D., Hu Y., Li L., Tian H., Chen Z., Wang L., Ma H., Yang H., Teng K. (2014). Highly pathogenic porcine reproductive and respiratory syndrome virus infection results in acute lung injury of the infected pigs. Vet. Microbiol..

[92] Zhao J., Wan S., Sun N., Sun P., Sun Y., Khan A., Guo J., Zheng X., Fan K., Yin W. (2021). Damage to intestinal barrier integrity in piglets caused by porcine reproductive and respiratory syndrome virus infection. Vet. Res..

[93] Guo H. (2022). Preparation of Monoclonal Antibody Against GP4 of PRRSVand Development of Candidate Strains for Replication-Deficient Vaccine. Master’s Thesis.

[94] Fu N., Zhou L., Chen M., Zhou L., Chen X., Dong N., Xiao C., Qiu Y., Li Z., Li B. (2026). Prokaryotic expression of porcine reproductive andrespiratory syndrome virus gp4 protein and preparationof polyclonal antibodies. Chin. J. Anim. Infect. Dis..

[95] Wang Y.P. (2018). Prokaryotic Expression Andidentification of Prrsvgp4 Protein. Master’s Thesis.

[96] Guo H., Wu D.G.W., Zhao H.Z., Liu C.Y., Hou L.N., Wang F.X., Wen Y.J. (2024). Prokaryotic Expression of PRRSV GP4 Protein and Preparation of MonoclonalAntibodies. Chin. J. Anim. Infect. Dis..

[97] Stadejek T., Oleksiewicz M.B., Potapchuk D., Podgórska K. (2006). Porcine reproductive and respiratory syndrome virus strains of exceptional diversity in eastern Europe support the definition of new genetic subtypes. J. Gen. Virol..

[98] Kimpston-Burkgren K., Correas I., Osorio F.A., Steffen D., Pattnaik A.K., Fang Y., Vu H.L.X. (2017). Relative contribution of porcine reproductive and respiratory syndrome virus open reading frames 2–4 to the induction of protective immunity. Vaccine.

[99] Fang Y., Snijder E.J. (2010). The PRRSV replicase: Exploring the multifunctionality of an intriguing set of nonstructural proteins. Virus Res..

[100] Wu W.H., Fang Y., Farwell R., Steffen-Bien M., Rowland R.R., Christopher-Hennings J., Nelson E.A. (2001). A 10-kDa structural protein of porcine reproductive and respiratory syndrome virus encoded by ORF2b. Virology.

[101] Zhou Y.J. (2005). Generation of Monoclonal Antibodies and Epitope Mapping for Structural Proteins of Porcine Reproductive and Respiratory Syndrome Virus. Ph.D. Thesis.

[102] Goldeck D., Perry D.M., Hayes J.W.P., Johnson L.P.M., Young J.E., Roychoudhury P., McLuskey E.L., Moffat K., Bakker A.Q., Kwakkenbos M.J. (2019). Establishment of Systems to Enable Isolation of Porcine Monoclonal Antibodies Broadly Neutralizing the Porcine Reproductive and Respiratory Syndrome Virus. Front. Immunol..

[103] Neumann E.J., Kliebenstein J.B., Johnson C.D., Mabry J.W., Bush E.J., Seitzinger A.H., Green A.L., Zimmerman J.J. (2005). Assessment of the economic impact of porcine reproductive and respiratory syndrome on swine production in the United States. J. Am. Vet. Med. Assoc..

[104] Liu G., Huang X., Yang Y., Chen M., Tian X., Song H., Wang H., Wang S., Wang H., Cai X. (2025). GP2a I118 and GP4 D43 play critical roles in the attachment of PRRSV to the CD163 receptor: Implications for anti-PRRSV infection targets. J. Virol..

[105] Zhang W., Wu A., Li H., He T., Dong Q., Zhang H., Chen J., Jiang S., Sheng J. (2025). Nanobodies Targeting the GP4 Protein Inhibit PRRSV Replication. Microorganisms.

[106] Corum M.R., Venkannagari H., Hryc C.F., Baker M.L. (2024). Predictive modeling and cryo-EM: A synergistic approach to modeling macromolecular structure. Biophys. J..

[107] Sun Y., Gao Y., Su T., Zhang L., Zhou H., Zhang J., Sun H., Bai J., Jiang P. (2025). Nanoparticle Vaccine Triggers Interferon-Gamma Production and Confers Protective Immunity against Porcine Reproductive and Respiratory Syndrome Virus. ACS Nano.

[108] Carlock M.A., Ross T.M. (2026). The evolution of influenza hemagglutinin: Structural numbering and receptor binding sites of influenza viruses capable of infecting humans. Front. Virol..

